# Perception-Based H.264/AVC Video Coding for Resource-Constrained and Low-Bit-Rate Applications

**DOI:** 10.3390/s25144259

**Published:** 2025-07-08

**Authors:** Lih-Jen Kau, Chin-Kun Tseng, Ming-Xian Lee

**Affiliations:** 1 Department of Electronic Engineering, National Taipei University of Technology, Taipei 106344, Taiwan; tsengchinkun@gmail.com (C.-K.T.); jackeyjay2002@yahoo.com.tw (M.-X.L.); 2 Tri-Service General Hospital Songshan Branch, Taipei 105309, Taiwan; 3 National Defense Medical Center, Taipei 114201, Taiwan

**Keywords:** H.264/AVC, Region of Interest (ROI), face detection, AdaBoost classifier, motion intensity, quantization parameter (QP)

## Abstract

With the rapid expansion of Internet of Things (IoT) and edge computing applications, efficient video transmission under constrained bandwidth and limited computational resources has become increasingly critical. In such environments, perception-based video coding plays a vital role in maintaining acceptable visual quality while minimizing bit rate and processing overhead. Although newer video coding standards have emerged, H.264/AVC remains the dominant compression format in many deployed systems, particularly in commercial CCTV surveillance, due to its compatibility, stability, and widespread hardware support. Motivated by these practical demands, this paper proposes a perception-based video coding algorithm specifically tailored for low-bit-rate H.264/AVC applications. By targeting regions most relevant to the human visual system, the proposed method enhances perceptual quality while optimizing resource usage, making it particularly suitable for embedded systems and bandwidth-limited communication channels. In general, regions containing human faces and those exhibiting significant motion are of primary importance for human perception and should receive higher bit allocation to preserve visual quality. To this end, macroblocks (MBs) containing human faces are detected using the Viola–Jones algorithm, which leverages AdaBoost for feature selection and a cascade of classifiers for fast and accurate detection. This approach is favored over deep learning-based models due to its low computational complexity and real-time capability, making it ideal for latency- and resource-constrained IoT and edge environments. Motion-intensive macroblocks were identified by comparing their motion intensity against the average motion level of preceding reference frames. Based on these criteria, a dynamic quantization parameter (QP) adjustment strategy was applied to assign finer quantization to perceptually important regions of interest (ROIs) in low-bit-rate scenarios. The experimental results show that the proposed method achieves superior subjective visual quality and objective Peak Signal-to-Noise Ratio (PSNR) compared to the standard JM software and other state-of-the-art algorithms under the same bit rate constraints. Moreover, the approach introduces only a marginal increase in computational complexity, highlighting its efficiency. Overall, the proposed algorithm offers an effective balance between visual quality and computational performance, making it well suited for video transmission in bandwidth-constrained, resource-limited IoT and edge computing environments.

## 1. Introduction

With the increasing deployment of Internet of Things (IoT) and edge computing technologies, video transmission over bandwidth-constrained networks and on resource-limited hardware platforms has become a pressing challenge. In such environments, how to ensure the perceptual visual quality of critical regions, such as human faces or areas where events occur, is of paramount importance. Therefore, perception-based video coding for low-bit-rate applications has emerged as an effective solution for resource-constrained systems like IoT nodes, smart surveillance cameras, and embedded video sensors [1,2,3,4,5,6,7,8]. While video coding standards have evolved rapidly over the past decade, from H.264/AVC [9] to HEVC [10] and, more recently, H.266/VVC [11], the associated computational complexity has increased substantially with each new generation. Although the newer standards offer better compression performance, their computational demands render them impractical for many real-time or low-power edge applications. In contrast, H.264/AVC remains the most widely adopted video compression standard in real-world applications due to its balance between efficiency and processing requirements [12,13,14]. Specifically, most practical video systems, including closed-circuit television (CCTV), video conferencing platforms (e.g., Zoom and Microsoft Teams), and mainstream streaming services (e.g., Netflix, YouTube, and Amazon Prime Video), continue to rely on H.264/AVC because of its compatibility with a wide range of devices and modest processing requirements.

To address the challenge of maintaining visual quality in critical areas under low-bit-rate constraints, perception-based rate control techniques have been proposed in the literature [1,2,3,4,5,6,7,8]. These techniques aim to allocate more bits to regions of interest (ROIs), such as areas with high motion or human faces, by leveraging characteristics of the human visual system. Studies have shown that humans are particularly sensitive to regions with high contrast, vivid color, motion, or semantically meaningful content (e.g., faces and moving objects). Therefore, focusing encoding resources on these areas leads to better subjective visual quality even when the overall bit rate budget is limited. In this paper, we focus on implementing a human visual perception-based rate control scheme for H.264/AVC to enhance the visual quality of perceptually significant regions in low-bit-rate scenarios. Although H.264/AVC is capable of achieving a good trade-off between bit rate and visual quality [12,13,14], quality degradation is still apparent in ROIs under severe rate constraints. In such cases, direct encoding or transcoding using standard H.264/AVC [9] often leads to unacceptable quality losses in perceptually important regions [15,16,17,18,19]. To address this issue, various ROI-based perceptual coding strategies have been developed [15,16,17,18,19,20,21,22,23,24,25,26,27,28,29,30,31], and we extend those efforts by proposing a lightweight, ROI-aware dynamic quantization parameter (QP) adjustment scheme that balances computational efficiency and visual quality enhancement and is well-suited for edge computing and IoT scenarios.

### 1.1. Literature Review

Perception-based video coding has attracted substantial research interest, particularly in the context of limited bandwidth and computational resources. This section categorizes recent studies according to their approaches and techniques in enhancing video quality through perceptual modeling, ROI detection, and resource-aware bit allocation strategies.

#### 1.1.1. Foundations of Perceptual Video Coding

The work in [17] presents a comprehensive overview of perceptual video compression, identifying three key components: perceptual model definition, coding implementation, and performance evaluation. Similarly, ref. [18] highlights the importance of integrating human visual system (HVS) characteristics into video communication engineering. The underlying mechanisms of visual attention and their modeling for image and video processing are further explored in [19].

#### 1.1.2. ROI Detection and Bit Allocation

In [20], a bit rate and computational power allocation scheme was proposed for conversational video applications. ROI was identified using direct frame difference and skin-tone detection, followed by adaptive QP adjustment to preserve perceptual quality. A region-based rate control approach was introduced in [21], where macroblocks (MBs) were grouped based on inter-frame rate-distortion (R-D) behaviors and encoded as basic units. This improved both subjective and objective quality. An ROI-based rate control algorithm was proposed in [22] using motion and texture analysis to generate saliency maps, allowing bit rate prioritization for perceptually important areas.

#### 1.1.3. JND-Based Perceptual Coding

To minimize perceptual distortion, ref. [23] proposed a perceptual framework utilizing just noticeable distortion (JND) metrics. A JND-normalized error model was incorporated into the rate-distortion optimization, enhancing compression without sacrificing visual quality. Similarly, ref. [24] introduced a rate control method based on structural similarity (SSIM), which was easily combined with perceptual mode decision techniques.

#### 1.1.4. Face-Centric and Attention-Driven ROI Coding

Obviously, human faces are critical ROIs in conversational video. Thus, a facial-feature-priority bit allocation method was proposed in [25]. Motion activity was first estimated using block mean differences to limit the search area for face detection. Then, face contours were refined and used to guide MB-level bit allocation. Additionally, ref. [26] introduced a foveated coding scheme leveraging audiovisual focus of attention. By analyzing the correlation between sound sources and visual signals, salient regions were prioritized based on their distance to the audio-emitting source.

#### 1.1.5. ROI-Based Transcoding and Mobile Adaptation

ROI-based transcoding methods for H.264/AVC were addressed in [27], using Bayesian models to determine ROI and establishing a bit rate conversion model through the curve fitting of non-zero transform coefficients. This allowed dynamic QP adjustment based on target bit rate and ROI weights. In [28], a temporal transcoding architecture was presented for mobile video communication. Frame selection was optimized based on visual complexity and temporal coherence, reducing bandwidth usage while maintaining perceptual quality.

#### 1.1.6. Real-World Applications and Frameworks

The integration of ROI-based video coding into practical systems was demonstrated in the VISNET/NAVSHP project [29], which used a ρ-domain linear rate-distortion model. The available bit budget was allocated based on ROI proximity and image complexity, enabling gradual quality degradation away from ROI. Visual rhythm analysis for automatic ROI detection was proposed in [30], employing multiple sampling lines to analyze motion and distinguish object versus camera dynamics. These studies collectively demonstrate the viability and effectiveness of perception-guided coding strategies in enhancing video quality, particularly under constrained bit rate and computation budgets typical of IoT and edge computing environments.

### 1.2. Motivation

In practical video surveillance systems deployed in IoT environments, transmission bandwidth is often limited and computing resources are constrained. For such conditions, the overall bit rate must be kept low, yet it remains critical to preserve the visual quality of important content. Specifically, regions that contain human faces or exhibit higher motion are of primary interest to both human observers and automated monitoring systems. Therefore, more coding bits should be allocated to these perceptually significant areas to maintain visual quality and interpretability. This paper proposes a perception-based dynamic QP adjustment strategy that prioritizes coding bits for ROIs under low-bit-rate settings using the H.264/AVC standard. The detection of human faces is performed using the lightweight Viola–Jones algorithm, which employs an AdaBoost-based cascade classifier to efficiently eliminate non-face regions in successive stages, thereby reducing computational complexity while maintaining real-time performance. This design choice ensures that the proposed system remains feasible for real-time applications on edge devices, where deep learning methods would otherwise be computationally prohibitive. For motion analysis, the average motion intensity across MBs in a video frame is compared with the individual MB’s motion vector to identify regions with higher motion. These identified ROIs, i.e., face regions and high-motion regions, are then assigned lower quantization parameters (QPs) to improve their visual quality, while background areas are encoded using higher QPs. A four-level QP adjustment scheme is applied accordingly, which is seamlessly integrated with the H.264/AVC rate control framework. To verify the effectiveness of the proposed strategy, the algorithm was tested on both JM9.8 and JM18.4 versions of the H.264/AVC reference software [32]. The experimental results demonstrate significant improvements in both subjective visual quality and objective PSNR within the identified ROIs, under the same bit rate constraint. Additionally, only a slight increase in computational complexity was observed, making the proposed solution highly practical for real-time, low-power video surveillance applications in IoT and edge computing environments. Some of the findings from this research were previously published in [33]. The present manuscript provides the full version of this study with thorough and comprehensive descriptions.

### 1.3. Innovation and Contribution of This Work

Just as introduced in Section 1.1 (Literature Review), region-of-interest (ROI)-based video coding has been extensively studied in the context of enhancing perceived video quality by prioritizing visually important regions, such as human faces, motion-intensive areas, or semantically meaningful objects. Existing methods typically fall into three broad categories: (1) traditional heuristics such as frame differencing and color-based skin detection, (2) saliency or attention modeling based on visual cues, and (3) deep learning-based semantic object detection using CNNs or transformers. In contrast, our approach is specifically designed for resource-constrained environments such as IoT or embedded edge devices, where computational and memory budgets are limited.

To facilitate a clear comparison between the proposed approach and existing ROI-based video coding strategies, Table 1 summarizes the key characteristics of traditional heuristic-based, saliency-driven, and deep learning-based methods, with an emphasis on their computational demands and suitability for real-time processing in IoT and edge environments. As shown, our method uniquely combines low computational complexity with practical feasibility in constrained environments, which sets it apart from other techniques. Overall, the main novelty and contributions of this paper are summarized as follows:

A. Dual-criteria ROI Definition for Perceptual Prioritization
  We propose a dual-criteria ROI detection strategy that identifies both (i) face regions and (ii) motion-intensive macroblocks (MBs) based on motion vector magnitude. This design ensures that regions most relevant to human visual perception receive enhanced visual quality, even under very low bit rate constraints.
B. Low-Complexity ROI Detection using Viola–Jones and Motion Analysis
  To maintain a lightweight system, we employed a Haar cascade classifier (Viola–Jones) for face detection and identified fast-moving MBs by comparing the current MB’s motion vector magnitude with the average motion vector of the previous frame. This strategy eliminates the need for computationally expensive saliency modeling or optical flow, making it well-suited for real-time applications on IoT and edge platforms.
C. Adaptive QP Control with Four-Level ROI Classification
  We developed a macroblock-level QP adjustment scheme based on a four-level ROI classification that allocates finer quantization to more perceptually sensitive regions. To further reduce visual artifacts such as blocking and maintain spatial consistency, we also introduced an inter-MB QP smoothness constraint.
D. System-Level Deployability and Encoder Integration
  We integrated our algorithm into the JM9.8 and JM18.4 versions of the H.264/AVC reference software and ensured its compatibility with the CPDT (Cascaded Pixel Domain Transcoder) frameworks. This facilitates seamless integration into both encoder and transcoder systems for real-world deployment.
E. Experimental Comparison between Viola–Jones Face Detection and Lightweight Deep Face Detector
  We conducted extensive experiments to compare our Viola–Jones-based detector with a MobileNetV1-SSD (INT8) deep learning model. We evaluated key performance metrics such as FLOPs, model size, memory usage, detection accuracy, and frame rate, including tests on Raspberry Pi 4 hardware. The results demonstrate that the Viola–Jones-based face detector offers a favorable trade-off between computational complexity and coding quality.
F. Improved Subjective and Objective Quality with Minimal Complexity Overhead
  Our run-time analysis reveals that the proposed method incurs only a 3–10% increase in execution time, while delivering notable improvements in both subjective visual perception and objective metrics (e.g., PSNR) within ROI areas. These benefits remain consistent even under ultra-low-bit-rate or bandwidth-limited scenarios, confirming the method’s practicality in resource-constrained environments.

**Table 1:** Comparison with Existing ROI-Based Methods.

Method Type	ROI Detection Technique	Computational Complexity	Real-Time Feasibility	Suitability for IoT Devices
Traditional Heuristics	Frame difference, skin-color detection	Low to Moderate	Moderate	Partial
Saliency-Based	Visual attention, saliency models (Itti-Koch, GBVS)	Moderate to High	Limited	No
Deep Learning-Based	CNNs (e.g., YOLO, SSD), semantic segmentation	High	No (requires GPU)	No
Proposed Method	Viola-Jones for faces + MV-based fast MBs	Very Low	Yes	Yes

In summary, the proposed ROI-based video coding approach is specifically designed to meet the constraints of low-bit-rate, low complexity environments. Its dual-ROI detection strategy and adaptive QP control scheme are particularly suitable for deployment on IoT devices and edge computing platforms, where real-time performance and efficient resource utilization are critical.

The remainder of this paper is organized as follows. Section 2 introduces human face detection using the AdaBoost classifier [34]. Section 3 provides a detailed description of the proposed perception-based visual quality enhancement algorithm with dynamic QP adjustment. Extensive experimental results, including both subjective and objective evaluations, are presented in Section 4, along with comparisons between the proposed method, the standard JM software, and existing state-of-the-art ROI-based visual quality enhancement techniques. Limitations of the proposed approach and potential directions for future work are discussed in Section 5. Finally, concluding remarks are provided in Section 6.

## 2. Human Face Detection with Adaptive Boosting Algorithm

It is well established that more bit resources should be allocated in regions of interest than in the background area during an encoding or transcoding process so that video content with better perceptual quality under limited bit resources can be generated. We can do this by selecting a smaller QP value for ROI areas and a larger QP value for background areas during the coding process. In this study, we aimed to allocate more bits to regions containing human faces and those exhibiting significant motion activities since these areas are most sensitive to the human visual perception system. In H.264/AVC coding or transcoding processes, the QP value was assigned for individual macroblocks. Therefore, we needed to determine which macroblocks contained human faces or larger motion activities. To determine the existence of an area containing human faces, we applied the well known adaptive boosting (so-called AdaBoost) classifier in this study [34].

In fact, face detection technology has advanced rapidly, with many AI-based algorithms being developed in recent years [35,36,37,38]. However, these AI-based techniques often require significant computational power, limiting their possibility to be applied in resource-constrained environments. Compared to AI-based algorithms, AdaBoost is preferred for its fast run-time performance, making it ideal for real-time applications like video streaming. It also requires minimal computational resources, making it suitable for devices with limited processing power, such as mobile phones, IoT devices, and embedded systems. Based on the above reasons, the AdaBoost face detector was employed in this study, allowing the proposed system to be easily deployed on edge computing units with limited resources.

### 2.1. Fundamentals of the AdaBoost Classifier

Adaptive boosting (AdaBoost) is a machine learning algorithm that is widely applied for classification applications and can be used in conjunction with many other learning algorithms [34]. The basic concept of AdaBoost is to use a large number of weak classifiers together to construct a strong classifier. In theory, as long as the ability of each classifier is slightly better than random prediction, i.e., their error rate is smaller than 0.5 for binary classification, the error probability of the final strong classifier will be close to zero when the number of weak classifiers approaches infinity [39]. Even classifiers with an error rate higher than 0.5 could be considered more useful than a random classifier since they will always have negative coefficients in the final linear combination of classifiers and, hence, behave like their inverses [39]. To adapt itself to the statistics of the data to be classified, AdaBoost is adaptive in the sense that subsequent classifiers built are tweaked in favor of those instances misclassified by previous classifiers, i.e., a misclassified data pattern will have a larger weight than that of correctly classified data samples [39].

In 2001, a visual object detection algorithm based on the AdaBoost algorithm in conjunction with a so-called integral image was proposed [40]. The object detection algorithm is capable of processing images in an extremely efficient manner and achieving high detection rates due to the following three contributions [40]. The first is the introduction of the “Integral Image” for image representation, which allows the feature extraction to be computed very quickly. The second is a learning algorithm based on AdaBoost with the use of a small number of simple visual features, called Haar-like feature rectangles, to form a set of extremely efficient classifiers. The third contribution is combining increasingly more complex classifiers in a cascade manner, which allows background regions of the image to be quickly discarded while spending more computation on promising, object-like regions [40].

**Figure 1:**
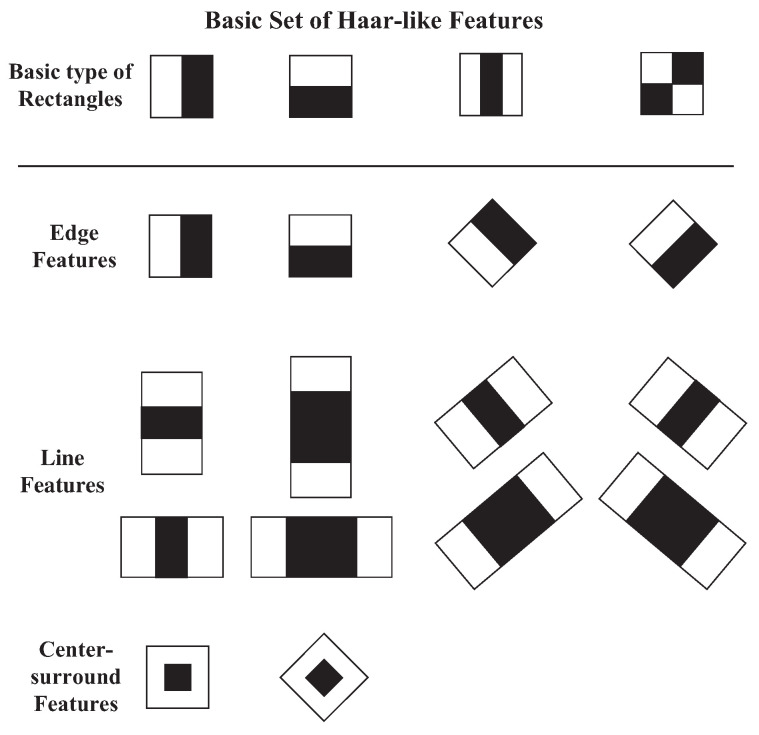
Basic and extended set of Haar-like feature rectangles [40,41,42].

To improve the accuracy of the object detection scheme based on a boosted cascade of simple feature classifiers, a set of rotated Haar-like features was proposed [41,42]. The basic feature rectangles and the extended set of Haar-like features are shown in Figure 1 [40,41,42]. As can be seen in Figure 1, the extended set of feature rectangles were obtained by stretching in horizontal and vertical directions or by rotation of the basic rectangles. The extended set of features rectangles significantly enriched the simple features for object detection and could be calculated efficiently [41,42]. To perform object recognition, usually, two or more feature rectangles are used simultaneously to form a feature or so-called weak classifier [41,42]. Moreover, the different shapes of feature rectangles or the same feature rectangle at different locations in an image are regarded as different weak classifiers. The feature value of a *j*th weak classifier composed of *N* feature rectangles is given by

(1) $$f_{j}=\sum_{i\in (1\ldots ,N)}w_{i}\times RectSum\left(r_{i}\right)$$

where *N* is the number of rectangles forming the *j*th weak classifier, $w_{i}$ is the weight corresponding to rectangle $r_{i}$, and $RectSum(r_{i})$ is the summation of gray levels in rectangle $r_{i}$ (i.e., the difference between the summation of gray levels in the white areas and those in the black areas in rectangle $r_{i}$).

In AdaBoost, each weak classifier just performs simple thresholding operations based on the feature value in (1) to determine if the area under evaluation is the target or not. These many weak classifiers are then trained by the AdaBoost algorithm based on their performance on classification correctness so that only a set of these are selected to form the final strong classifier [41,42].

### 2.2. Face Detection with the OpenCV AdaBoost Classifier

Based on the introduction in the previous subsection, lots of procedures, e.g., a large number of positive and negative training samples, different kinds of and representative Haar-like features, and enough and efficient training cycles, are required before a well-trained AdaBoost classifier can be obtained. Fortunately, there are well-trained classifiers that are available publicly, e.g., the Open Source Computer Vision Library (OpenCV). The OpenCV was developed by an open source project with C/C++ language, initiated and supported by INTEL corporation since 1999 [43]. With its long-term accumulation of image databases, a number of different and well-trained classifiers are available in the OpenCV “haarcascades” folder, such as the human mouth, nose, body, and other classifiers for different purposes, including the human face classifier “haarcascade_frontalface_alt.xml” [43]. As these classifiers in OpenCV have been successfully implemented in many applications, in this study, we used an AdaBoost classifier with OpenCV for human face detection.

**Figure 2:**
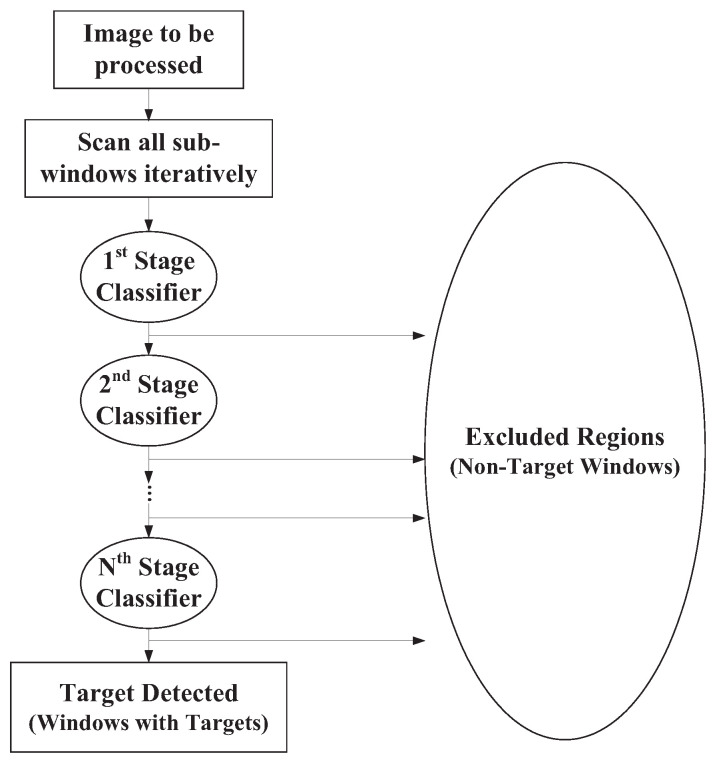
Hierarchy of a cascaded classifier.

The architecture of the AdaBoost face detector in OpenCV is shown in Figure 2. As can be seen in Figure 2, the face detector was composed of 22 stages in a cascade manner. Furthermore, each stage in Figure 2 was a strong classifier, i.e., each stage classifier was composed of a set of weak classifiers. For example, the first stage was composed of three weak classifiers, and the 22nd stage (the final stage) was composed of 212 weak classifiers. To speed up the efficiency of the classification process, the early stages were composed of simple and important features, which allowed a large number of negative samples to be quickly excluded from being regarded as the target. Those complex features were then placed in later stages. With the cascading architecture, the face detection efficiency could be significantly enhanced [40,41,42,43].

To apply the face detection mechanism with OpenCV, some of the important functions are listed below:

- CascadeClassifier face_cascade //define a variable with type classifier
- String face_cascade_name=“haarcascade_frontalface_alt.xml” //Declare the use of a frontal face classifier
- face_cascade.load (face_cascade_name) //Read in the declared classifier
- face_cascade.detectMultiScale (frame_gray, faces, 1.1, 2, 0 | CV_HAAR_SCALE_IMAGE, Size (30, 30)) //Perform face detection with a minimal size of 30×30 pixels
- faces[*i*] //Center position of the detected *i*th face
- faces[*i*].width //Width of the detected *i*th face
- faces[*i*].height //Height of the detected *i*th face

**Figure 3:**
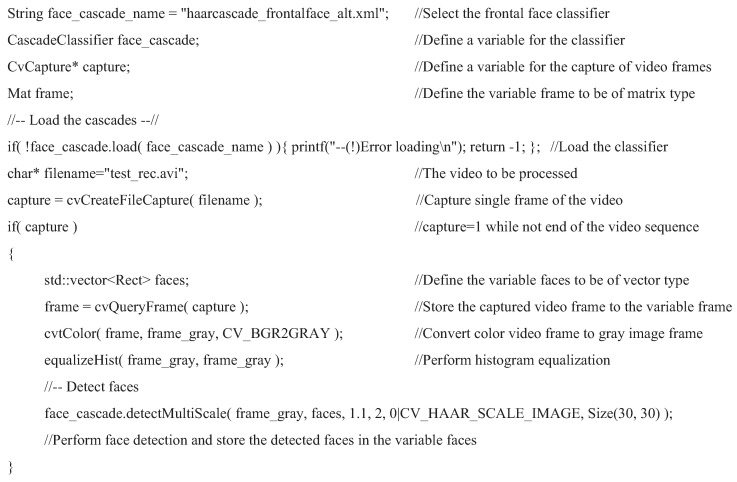
Pseudo code for AdaBoost face detection with OpenCV.

In OpenCV, the face detection process was performed via the function “face_cascade.detectMultiScale”. The smallest face area that could be detected was set to 30×30 pixels in this study. The detected face position as well as the width and height were recorded for the variables “faces[i]”, faces[i].width, and faces[i]. height, respectively. Instead of using the value of “hue”, i.e., the color tone of skin, as one of the features for human face detection, the color video frames were captured and transformed to gray-scale images for classification in a sequential manner with the OpenCV face classifier. With this process, the face detection result were independent of the color (or hue) of the skin. The experimental results for human face detection with OpenCV can be found in Section 4. To summarize, a pseudo code for applying the face detection mechanism with OpenCV is shown in Figure 3.

## 3. Proposed Perception-Based Visual Quality Enhancement Algorithm

In this section, the proposed perception-based QP control algorithm will be introduced in a detailed manner. The proposed algorithm can be easily applied in either H.264/AVC coding or transcoding systems. The main idea is to allocate more bits to the regions of interest, i.e., macroblocks with human faces or with larger motion activities, than in background areas under limited bit resources. In the H.264/AVC coding system, the optimal trade-off between visual quality and the target bit rate is attained via a complex rate distortion optimization (RDO) process so that the prediction mode, block size, and quantization parameter for individual macroblocks can be determined [12,13,14].

For the proposed algorithm, we mainly concentrated on the quality enhancement of P-frames in a video sequence. To locate the macroblocks in our regions of interest, the OpenCV “frontalface” classifier was applied for face detection and the the motion vector of individual macroblocks was retrieved for motion activity analysis. The rate control and rate optimization mechanism in the H.264/AVC coding system were all enabled in the proposed algorithm [32].

In the H.264/AVC standard, a quantization parameter (QP_*rate_control*_) was assigned to each macroblock [12,13,14]. We then determined if the QP_*rate_control*_ needed to be adjusted by checking if the macroblock was in our regions of interest. If the macroblock to be encoded was in our regions of interest, a smaller quantization parameter than the QP_*rate_control*_ was given, depending upon the proposed QP adjustment algorithm, so that better perceptual visual quality could be obtained [12,13,14,32]. In this study, the macroblock to be encoded was divided into the following four kinds of types.

- Type 3: MB with both human faces and larger motion activities.
- Type 2: MB with human faces.
- Type 1: MB with greater motion activities.
- Type 0: Background MBs, i.e., MB with neither human faces nor larger motion activities.

It was reasonable to allocate more bits for a macroblock of higher type than for that of a lower type. In other words, a smaller QP was assigned for a macroblock of a higher type, and vice versa. The proposed dynamic QP adjustment strategy for the four types of macroblocks will be explained in the following subsections.

### 3.1. ROI with Human Faces

In this subsection, the dynamic QP adjustment strategy for macroblocks with human faces will be introduced in a detailed manner. For explanation convenience, we use the quarter common intermediate format (QCIF) video sequence “Foreman” as an example. To record if an MB was within the area of human faces, the QCIF video frame (144×176) was divided into 9×11 MBs and then the type of each MB was recorded in a 9×11 matrix $Q_{Face\_ROI}$.

**Figure 4:**
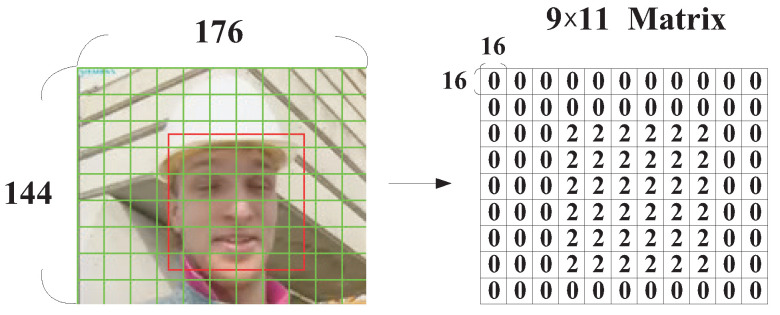
Macroblocks that are within parts of human faces in a video frame.

To determine the region that contained human faces, the OpenCV “frontalface” classifier was applied. As can be seen in Figure 4, the face areas detected by the AdaBoost algorithm were enclosed by a rectangle with a red line boundary, while the green lines denote the boundaries between MBs. After the face areas were determined, the elements in the 9×11 matrix $Q_{Face\_ROI}$ for those MBs that were within the area of human faces were recorded with a value of 2, while the others (background areas) were given a value of 0 (as in Figure 4), i.e.,

(2) $$Q_{Face\_ROI}\left(x,y\right)=\left\{\begin{array}{cc} 2, & \mathrm{MB}\;\mathrm{with}\;\mathrm{frontal}\;\mathrm{face};\\ 0, & \mathrm{ow}, \end{array}\right.$$

where (x,y) denotes the position of an MB in the video frame.

### 3.2. ROIs with Greater Motion Activities

**Figure 5:**
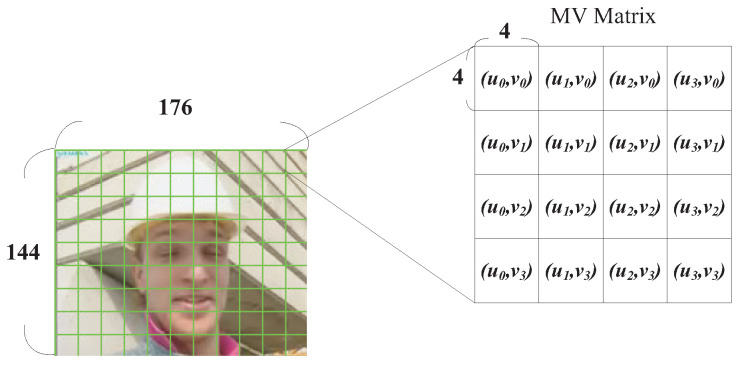
Structure for recording motion vectors with a 4×4 matrix in a macroblock.

In addition to those regions containing human faces, areas with greater motion activities are also quite sensitive to human visual perception systems. More bits should be allocated to make motion activity appear smoothly in consecutive video frames. To locate the MBs with greater motion activities, the motion vector strength of individual MBs was analyzed. In the H.264/AVC coding standard, the smallest block size that can be used for motion estimation is 4×4. Therefore, H.264/AVC records the motion vector with a basic unit of every 4×4 pixels in an MB (Figure 5). In this study, the sixteen motion vectors in an MB were recorded in a 4×4 matrix, as shown in Figure 5, where *u* represents the horizontal and *v* represents the vertical direction of the motion vector, respectively. For explanation convenience, we use the QCIF video sequence “Foreman” in Figure 5 as an example.

**Figure 6:**
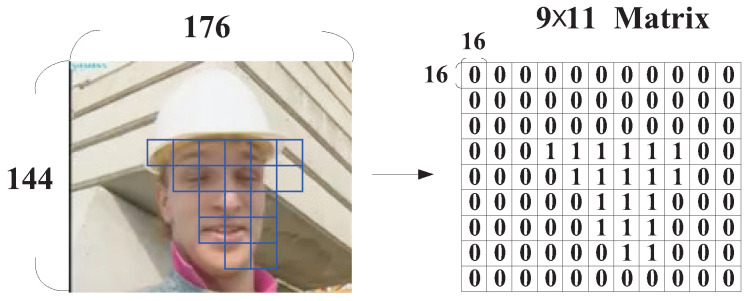
MBs with larger motion intensity in a test video frame.

To determine if an MB was with greater motion activity or not, we first calculated the motion intensity $MI^{t}$ in each MB according to the following equation:

(3) $$MI^{t}\left(x,y\right)=\sum_{k=0}^{3}\sum_{l=0}^{3}\sqrt{{\left(u_{k}\right)}^{2}+{\left(v_{l}\right)}^{2}},$$

where the superscript *t* denotes the frame number and $\left(u_{k},v_{l}\right)$ denotes one of the sixteen motion vectors inside the macroblock at position (x,y). We then defined the average motion intensity $MI_{Avg}^{t}$ of a video frame as below (using QCIF as an example):

(4) $$MI_{Avg}^{t}=\frac{1}{9\times 11}\sum_{x=0}^{8}\sum_{y=0}^{10}MI^{t}\left(x,y\right),$$

where $MI^{t}\left(x,y\right)$ is the motion intensity of the MB at position (x,y). We could then determine if an MB at position (x,y) had greater motion activity by checking if the first condition in the following inequality held:

(5) $$Q_{MI\_ROI}\left(x,y\right)=\left\{\begin{array}{cc} 1, & \frac{MI^{t}\left(x,y\right)}{MI_{Avg}^{t-1}}\geq \theta ;\\ 0, & \mathrm{ow}, \end{array}\right.$$

where $Q_{MI\_ROI}$ denotes the matrix that records if the MB under evaluation has greater motion activity or not, θ is a threshold and was set to be 2.5 in this study, $MI^{t}\left(x,y\right)$ is defined in (3), and $MI_{Avg}^{t-1}$ is defined in (4), but with superscript time index replaced by t−1, i.e., we calculated the average motion intensity of the preceding reference video frame. As can be seen in (5), an MB was regarded as having greater motion activity if its motion intensity $MI^{t}\left(x,y\right)$ was greater than or equal to 2.5 times the average motion intensity $MI_{Avg}^{t-1}$. Moreover, a value of 1 was given and stored in the corresponding element of a 9×11 matrix $Q_{MI\_ROI}$ if the MB was regarded as having greater motion activity; otherwise, the corresponding element was assigned a value of 0 (as in Figure 6).

It should be noted that the threshold 2.5 for the determination of greater motion activity was chosen empirically. We conducted tests in our experiments with a threshold greater or smaller than 2.5. Obviously, a larger portion of MBs was regarded as having greater motion activity with a smaller threshold (smaller than 2.5), and this had a negative impact on the perceptual visual quality enhancement since the limited bit resources could not be concentrated on those MBs in our regions of interest. On the contrary, only a few MBs were regarded as part of the ROI, and those MBs that did not exceed the threshold but that had greater and continuous motion activity degraded the perceptual visual quality in consecutive video frames if a larger threshold than 2.5 was used. Based on these observations, we selected the threshold to be 2.5 and found it worked very well in this study.

### 3.3. Proposed Dynamic QP Adjustment Strategy

**Figure 7:**
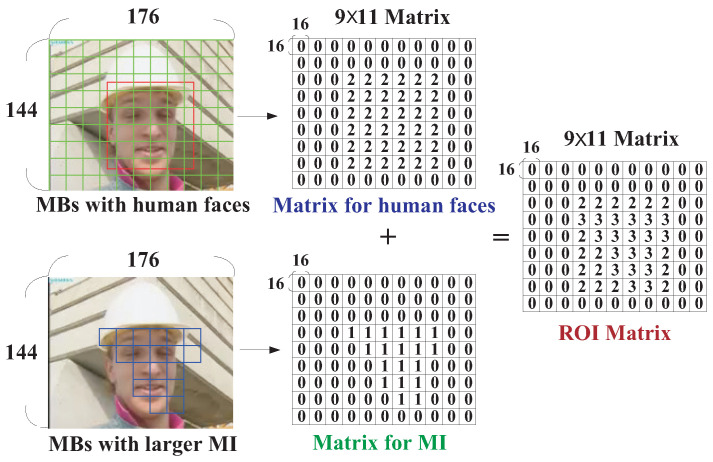
Matrix for QP adjustment in a video frame.

After the matrices, i.e., $Q_{Face\_ROI}$ and $Q_{MI\_ROI}$ recording MBs containing human faces and with greater motion activities, were obtained in (2) and (5), we could then determine the QP adjustment strategy by summing up the two matrices ($Q_{Face\_ROI}$ and $Q_{MI\_ROI}$) to obtain a *weighted ROI matrix* ${\mathrm{WQ}}_{ROI}$ as below:

(6) $${\mathrm{WQ}}_{ROI}=Q_{Face\_ROI}+Q_{MI\_ROI},$$

where $Q_{Face\_ROI}$ and $Q_{MI\_ROI}$ are defined in (2) and (5), respectively. To illustrate the idea of the proposed dynamic QP adjustment strategy, the process of obtaining the ${\mathrm{WQ}}_{ROI}$ matrix is best illustrated in Figure 7. With this process, the elements in the matrix ${\mathrm{WQ}}_{ROI}$ could have four kinds (types) of values, i.e., 0 to 3, indicating the importance of the MB. Actually, the values were in compliance with the four types defined at the beginning of this section, indicating the content type of the MB.

As can be seen in Figure 7, an element with a value of 3 in the weighted ROI matrix ${\mathrm{WQ}}_{ROI}$ (rightmost matrix of Figure 7) indicated that both part of a human face and greater motion activity were in the MB simultaneously. Therefore, a highest quality with a smaller QP needed to be given for such kinds of MBs in response to the presence of a face image region and a greater tendency to move. In this study, the QP for individual MBs was adjusted depending upon the corresponding value in ${\mathrm{WQ}}_{ROI}$. Instead of assigning a fixed QP to MBs of the same type, we adjusted the QP of individual MBs dynamically. To do this, recall that an initial quantization parameter called QP_*rate_control*_ was assigned for individual MBs dynamically if the rate control mechanism was enabled during the H.264/AVC coding process. We then assigned MBs of type 3 a final quantization parameter (QP) three levels lower than the QP_*rate_control*_ originally given by the rate control mechanism, i.e., ${\mathrm{QP}}_{\mathrm{Type}\;3}={\mathrm{QP}}_{rate\_control}-3$. Similarly, a value of 2 in the weighted ROI matrix ${\mathrm{WQ}}_{ROI}$ indicated that aa face region was detected in the MB but that the intensity of motion activity was relatively low. For this case, a second highest quality needed to be given, and a final QP that was two levels lower than the QP_*rate_control*_ was assigned for type 2 MBs, i.e., ${\mathrm{QP}}_{\mathrm{Type}\;2}={\mathrm{QP}}_{rate\_control}-2$. The element of an MB with greater motion activity but that did not belong to any human face region was assigned a value of 1 in the weighted ROI matrix ${\mathrm{WQ}}_{ROI}$ (i.e., type 1 MB). For type 1 MBs, a final QP one level lower than the QP_*rate_control*_ was assigned, i.e., ${\mathrm{QP}}_{\mathrm{Type}\;1}={\mathrm{QP}}_{rate\_control}-1$. Finally, a value of 0 denoted the case of video background. Due to the continuity and unity characteristics of a background, we did not have to allocate excessive coding bits to such areas. Therefore, the QP was kept unaltered, i.e., ${\mathrm{QP}}_{\mathrm{Type}\;0}={\mathrm{QP}}_{rate\_control}$.

**Figure 8:**
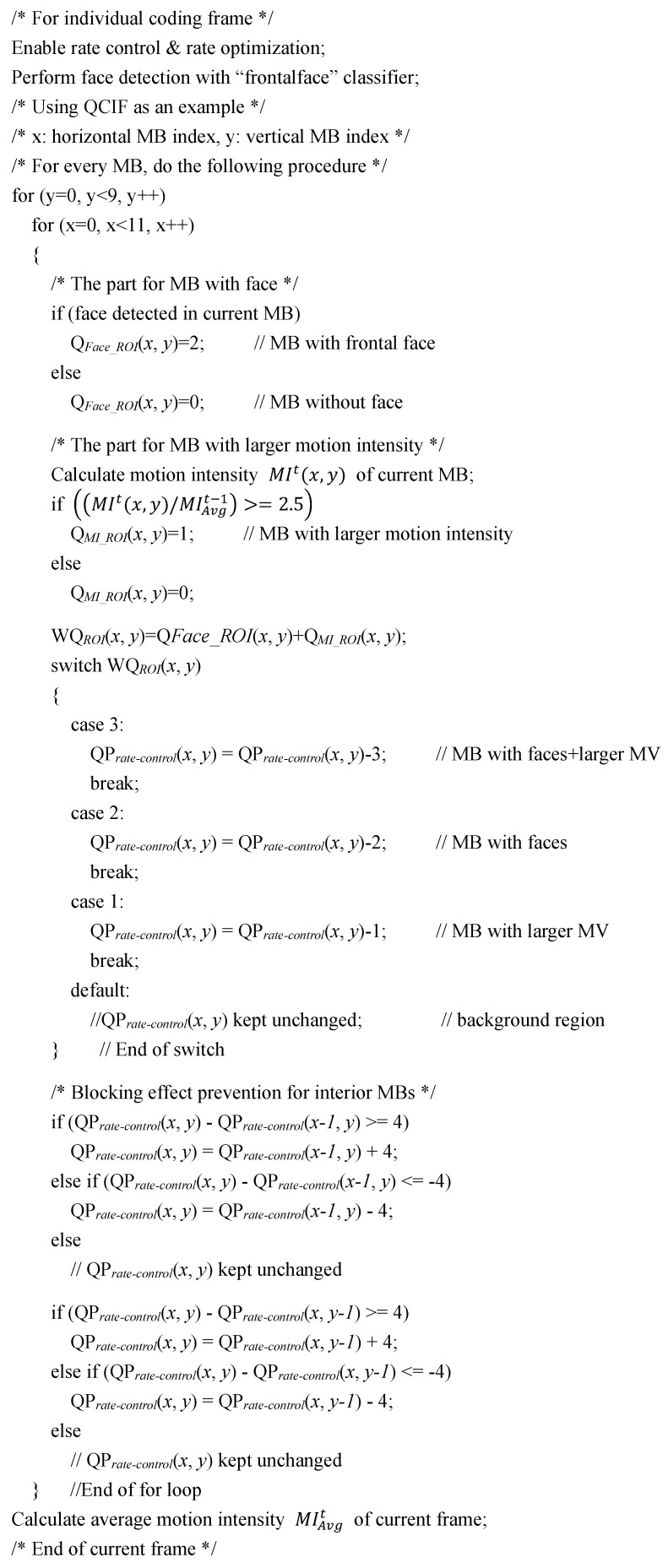
Pseudo code of the proposed dynamic QP adjustment strategy.

So far, we have one more thing to notice, which is to avoid incurring the so-called “blocking effect” during the adjustment process of QPs. The major cause of the blocking effect is due to a larger difference of the QP assigned between neighboring MBs. To avoid incurring the blocking effect, the QP assigned for consecutive MBs could differ only to a certain extent. In this study, the differences of the QP assigned for the coding MB and that of its left as well as its top MBs were confined to four levels, i.e., the QP assigned between neighboring MBs could not differ by more than four levels to keep the neighboring MBs visually continuous. To summarize, the pseudo code of the proposed perception-based dynamic QP adjustment strategy is shown in Figure 8.

### 3.4. Applying the Proposed Algorithm to H.264/AVC Transcoding Systems

**Figure 9:**
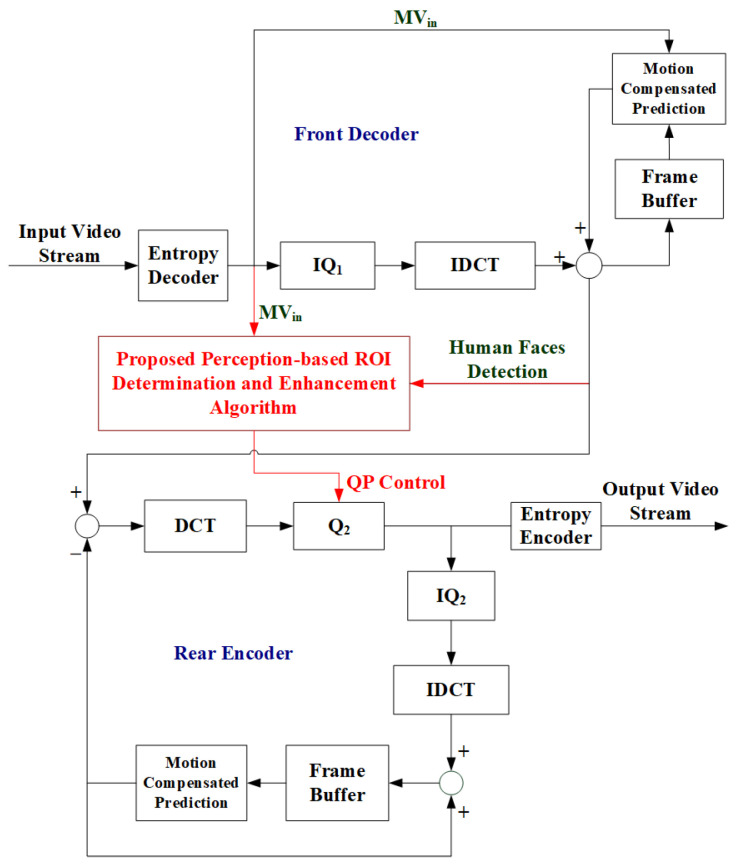
Block diagram of a Cascaded Pixel-Domain Transcoder (CPDT).

It is noted that the proposed algorithm can also be applied to H.264/AVC transcoding systems. In general, a video transcoder can be used to transfer a higher-bit-rate video stream to a lower-bit-rate sequence to cope with the insufficient channel bandwidth or insufficient computation capability of the receiver devices. To achieve this goal, a video transcoder was composed of a front-end decoder (front decoder) and a back-end encoder (rear encoder). The front-end decoder was mainly composed of a H.264/AVC bit stream decoder, while the back-end encoder just performed parts of or all of the H.264/AVC video coding procedures to meet the preferred target bit rate requirements. Based on the procedures performed in the front-end decoder and the back-end encoder, the transcoder could be further divided into two kinds of commonly used architectures: the Cascade Pixel Domain Transcoder (CPDT) and Cascade DCT Domain Transcoder (CDDT) [15,16]. In CPDT, the video frames were rebuilt completely in the front-end decoder, and we could then apply the AdaBoost frontalface classifier to check if any human face existed in a video frame (Figure 9). It should be noted that the proposed algorithm could not be applied in the CDDT architecture since the front-end decoder did not perform inverse discrete cosine transform (DCT) after the inverse quantization process, and we could not obtain the reconstructed video frame for human face detection in CDDT. Figure 9 gives a block diagram showing the fusion of the proposed perception-based ROI enhancement algorithm with CPDT. As can be seen in Figure 9, the motion vector as well as the whole video frame could be retrieved after the entropy decoder and the inverse DCT with motion compensation process. After those MBs with human faces and greater motion activities could be determined, the QP for the rear encoder could be decided upon (as the dashed arrow points to the block Q_2_), so that better visual quality in the regions of interest could be achieved under limited bit resources.

## 4. Experiments

In this section, subjective and objective visual quality evaluations of regions of interest under certain lower target bit rates will be described to highlight the usefulness of the proposed approach. Moreover, we also compare the proposed algorithm with the existing ROI-based H.264/AVC encoder in [20] by using the widely applied objective metric PSNR. For fair comparisons, we implemented and executed the proposed approach on the same version of JM software and all the parameter settings were identical as those in [20]. The results obtained by using the proposed approach are then compared with that obtained by standard JM reference software under identical parameter settings as those in [20]. Finally, as the proposed algorithm was implemented based on the JM software, we also show in this study the execution time of the proposed approach for different test video sequences under various target bit rates with JM9.8 as well as with the latest version JM18.4 to obtain a picture of the run-time performance of the proposed approach [32].

The test sequences, image resolutions, and corresponding bit rate settings used for the performance evaluations are summarized in Table 2.

As shown in Table 2, a range of target bit rates, specifically 8 kbps, 11 kbps, 19 kbps, 22 kbps, 64 kbps, 68 kbps, 118 kbps, and 244 kbps, were employed, covering both ultra-low-bit-rate scenarios (e.g., 8 kbps and 11 kbps) and higher rates of up to 244 kbps. These specific configurations, including image resolutions and test sequences, were carefully selected to enable fair comparisons with prior studies and state-of-the-art video encoders that adopted the same target bit rate constraints and used identical evaluation sequences.

### 4.1. Face Detection by Adaboost Algorithm

**Figure 10:**
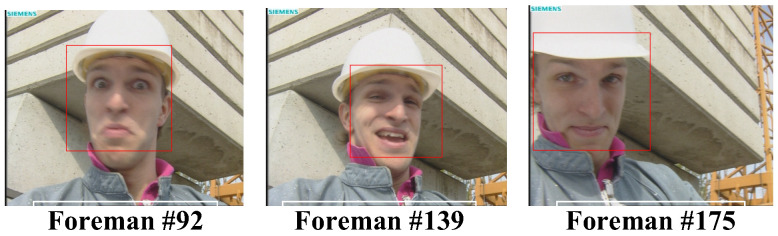
Face detected in the test video “Foreman”.

**Figure 11:**
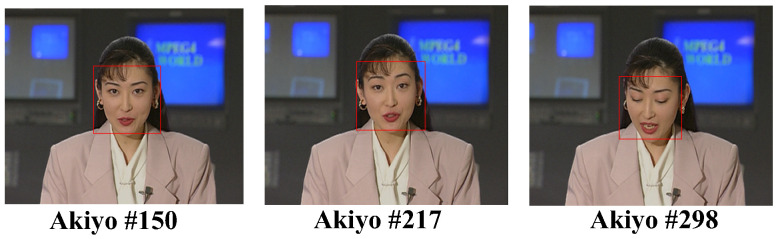
Face detected in the test video “Akiyo”.

In this subsection, the performance of human face detection with the AdaBoost frontalface classifier will be described. The three common intermediate format (CIF) video sequences, “Foreman”, “Akiyo”, and “Mother-Daughter”, as well as the one quarter common intermediate format (QCIF) video sequence, “Miss America”, were used as the test video sequences. For the CIF test video sequence “Foreman” in Figure 10, the detected face regions were enclosed by a rectangle with a red line border. As can be seen in Figure 10, the face area of the foreman could be correctly identified, no matter the facial expression or captured point of view. Figure 11 shows the results of another CIF test video sequence “Akiyo”. As can be seen in Figure 11, the face region could be successfully recognized for a variety of different facial expressions and captured points of view, and even with closed eyes.

**Figure 12:**
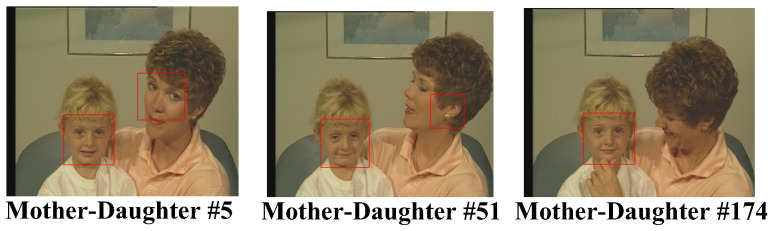
Face detected in the test video “MotherDaughter”.

**Figure 13:**
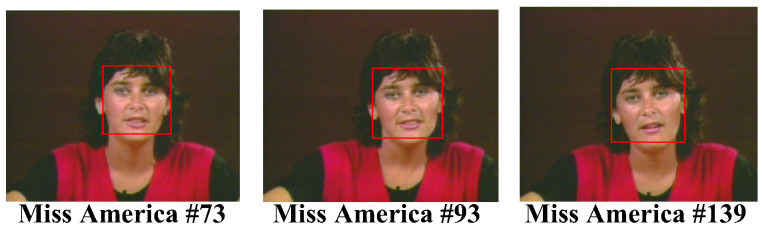
Face detected in the test video “MissAmerica”.

For the CIF test video sequence “Mother-Daughter”, we see in Figure 12 that the face region could be successfully detected in most of the cases, even when two faces existed in the same video simultaneously. The only one that failed to be recognized was the mother’s face area in frame number 174, where the mother turned her face to the right, facing her daughter, and, thus, few frontal face features could be acquired for face recognition. Finally, we investigated the AdaBoost algorithm with the QCIF test video sequence “Miss America”. The QCIF video format had a smaller dimension than that of the CIF video format. The results of the test video are shown in Figure 13. As can be seen in Figure 13, the face region of miss America could be correctly recognized even with the video being of a smaller resolution or dimension.

### 4.2. Face Detection Complexity: Haar Cascade vs. MobileNet-SSD

The rapid advancements in artificial intelligence and deep learning technologies in recent years have led to the development of numerous face detection algorithms and models. Among them, MobileNet has attracted attention for its lightweight architecture, making it suitable for deployment on embedded systems or edge computing units with limited computational resources. Building on this context, we conducted a fair comparison between two face detection algorithms, the traditional Haar cascade classifier (”haarcascade_frontalface_alt.xml”) provided by OpenCV and the MobileNetV1-SSD (INT8) approach with a MobileNetV1 backbone [44,45]. Key performance metrics assessed included computational complexity (FLOPs), model size, memory consumption, inference speed (FPS), detection rate, precision, recall, platform compatibility, and suitability for edge computing applications.

The MobileNetV1-SSD (INT8) model utilized in this comparison accepted an input resolution of 300 × 300 pixels, as specified in the official TensorFlow Lite model repository. In contrast, the Viola–Jones Haar cascade classifier processed images at a CIF resolution of 352 × 288 pixels. The evaluation was conducted on the same platform (Raspberry Pi 4 Model B, 8 GB RAM) and the performance metrics were assessed using the FDDB dataset [46], which contained 2845 images with 5171 labeled faces. For fairness, all metrics such as precision and recall were computed using a commonly accepted Intersection over Union (IoU) threshold of 0.5. It should be noted that the detection rate reported for Viola–Jones was based on face-level detection without enforcing a strict IoU threshold (i.e., any overlapping bounding box is considered a correct detection). As such, the detection rate could appear to be higher than recall, which applied a stricter IoU ≥ 0.5 criterion. The comparative results are summarized in Table 3.

As shown in Table 3, the MobileNetV1-SSD (INT8) detector significantly outperformed the traditional Viola–Jones (Haar) classifier across most evaluation metrics, particularly in detection accuracy. At an IoU threshold of 0.5, MobileNet achieved higher precision (0.87) and recall (0.85) compared to those achieved by the Viola–Jones classifier. In terms of computational load, however, Viola–Jones remained more lightweight, with a model size under 1 MB and only 2 M FLOPs, making it more suitable for ultra-low-power or legacy systems where deep learning inference engines may not be feasible. In contrast, MobileNetV1-SSD (INT8) required significantly more computational resources (570 M FLOPs and 6.8 MB in size), but the increased complexity was justified by its robust detection performance across varied facial poses. The inference speed (FPS) on Raspberry Pi 4 also reflected this trade-off: Viola–Jones offers an average frame rate of 15 FPS, suitable for real-time monitoring with frontal faces, whereas MobileNetV1-SSD (INT8) runs at 8 FPS on average but offers greater flexibility in real-world scenarios, including partial occlusions, side faces, and varying lighting conditions.

In conclusion, the traditional Haar cascade classifier remains a practical and efficient choice for resource-constrained environments like embedded IoT systems due to its significantly lower computational complexity and memory requirements. However, if detection robustness and accuracy are the priorities, and the system can support a moderate increase in computational cost, MobileNetV1-SSD (INT8) presents a more reliable and scalable alternative.

### 4.3. Subjective and Objective ROI Visual Quality Evaluation

This subsection presents subjective and objective visual quality comparisons with the standard JM software, focusing on ROIs containing human faces. To do this, we implemented the proposed algorithm on JM9.8, with the parameter settings identical to those in [20]. The same set of parameters will be used again for fair comparisons with the results reported by [20] in the subsequent subsection. In this subsection, the three CIF test video sequences, “Foreman”, “Akiyo”, and “Mother-Daughter”, will be used for subjective and objective ROI quality evaluation. The settings of the proposed approach on JM9.8 are listed below.

01. RD Optimization: on
02. Entropy coding: CABAC
03. SearchRange: 16
04. NumberReferenceFrames: 2
05. NumberBFrames: 0
06. RateControlEnable: 1
07. InitialQP: 30
08. BasicUnit: 1

**Figure 14:**
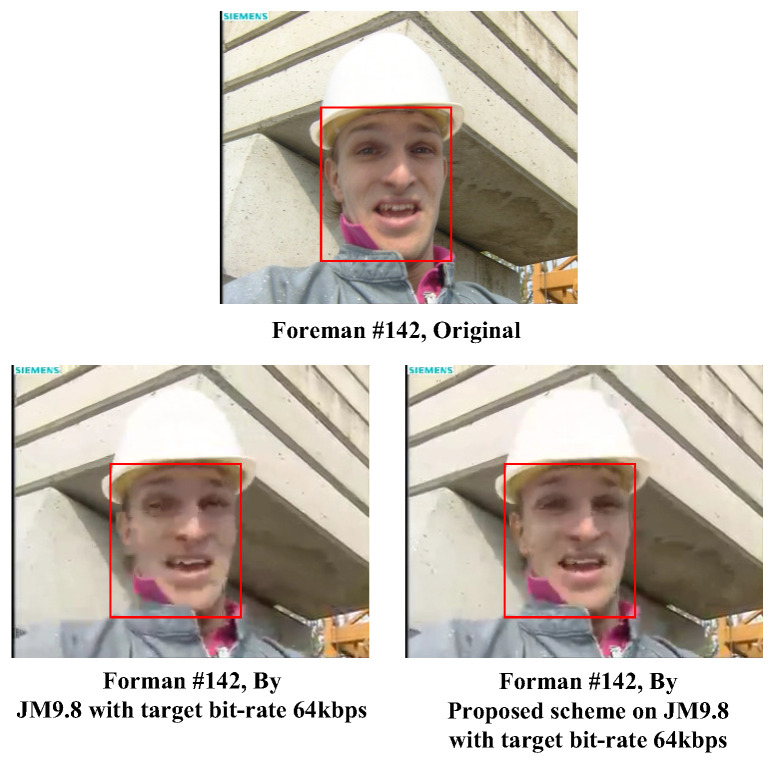
Subjective visual quality evaluation for the test video frame “Foreman”.

The comparison result between the proposed approach and that obtained by using the standard JM9.8 for the test video “Foreman” with frame number 142 under a target bit rate of 64 kbps is shown in Figure 14. For comparison purposes, we also show in the top area of Figure 14 the original image of frame number 142. As can be seen in Figure 14, the image obtained by using the proposed approach had a distinct and better contour around the eyes (especially the right eye) of the foreman than that obtained by using the standard JM9.8. Furthermore, a finer contour on the left side of his face could also be observed with the proposed approach. To look further into our regions of interest, we can see in Figure 14 the two sub-images enclosed by rectangles with a red line border. A significantly better visual quality on ROIs with human faces could be easily observed with the proposed approach since limited bit resources were concentrated and allocated on MBs containing human faces and greater motion intensities. Indeed, the PSNRs of the ROI sub-images in Figure 14 were 30.212 dB (proposed) and 22.368 dB (standard JM9.8), respectively, which demonstrates the superiority of the proposed approach over standard JM reference software.

**Figure 15:**
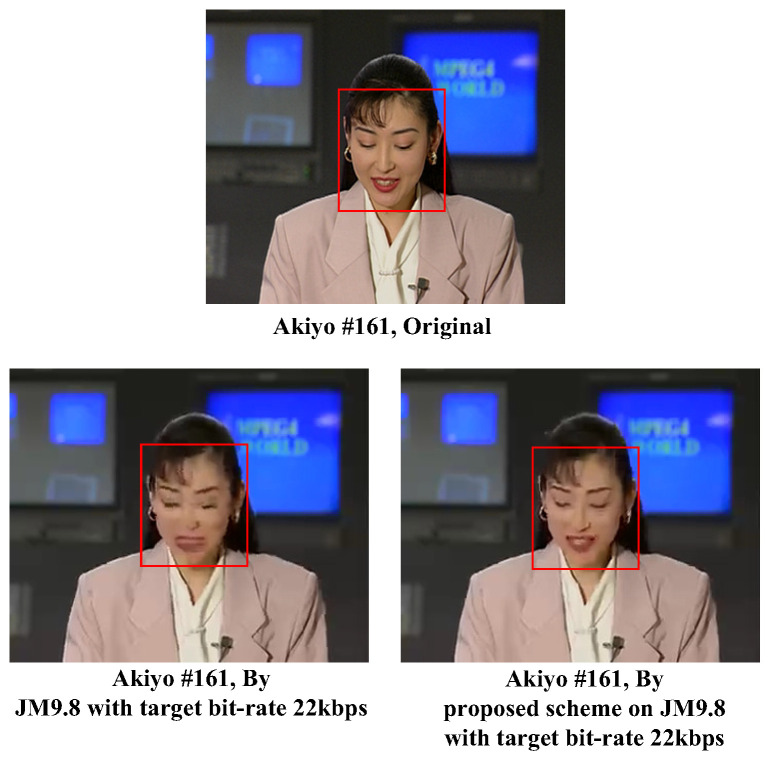
Subjective visual quality evaluation for the test video frame “Akiyo”.

Figure 15 shows the original image and comparison results of the proposed approach and that of standard JM9.8 for the CIF test video “Akiyo” with frame number 161 under a very low target bit rate of 22 kbps. To look further into the ROI, we can see in Figure 15 the two sub-images enclosed by rectangles with a red line border. When compared to the result obtained by the standard JM9.8, we see that significantly better visual quality could be obtained for the contour around her eyes and lips with the proposed approach. The better visual quality of the proposed approach over that of standard JM reference software for ROIs could be best observed by using the widely applied objective metric PSNR. Actually, the PSNRs of the two sub-images at the bottom of Figure 15 were 27.216 dB (proposed) and 25.956 dB (standard JM9.8), respectively.

**Figure 16:**
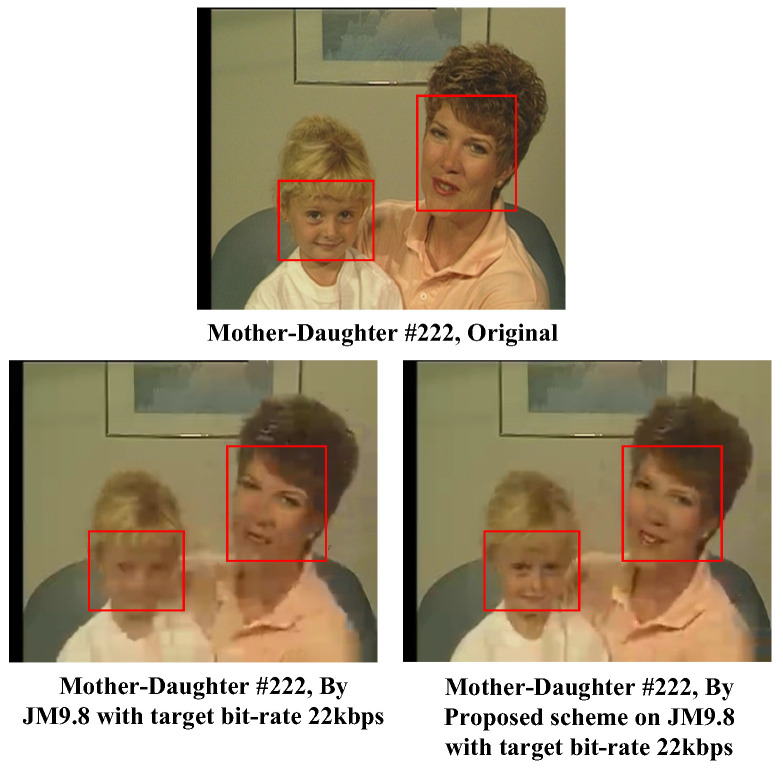
Subjective visual quality evaluation for the test video frame “MotherDaughter”.

Finally, the comparison results of the CIF test video “Mother-Daughter” with frame number 222 under a very low target bit rate of 22 kbps are shown in Figure 16. We first examined the result of the ROI obtained by using the standard JM9.8 software. As can be seen in the bottom left image of Figure 16, i.e., the result obtained by standard JM software, the contours around the mother’s eyes as well as her lips were hard to recognize, and the daughter’s face was almost illegible. On the other hand, we can see in the bottom right image of Figure 16 that a significant visual quality improvement could be obtained for the face areas of the mother and her daughter. The face contours of the daughter were apparent, and the contours around the mother’s eyes and lips were distinct using the proposed approach. Actually, the PSNRs of the two sub-images at the bottom of Figure 16 containing the face area of the mother were 30.028 dB (proposed) and 29.948 dB (standard JM9.8), respectively, and those of the image containing the face area of the daughter were 30.578 dB (proposed) and 28.492 dB (standard JM9.8), respectively. Obviously, the superiority of the proposed approach over standard JM reference software under the condition of a very low bit rate could be demonstrated again in this experiment. To highlight the performance of the proposed approach, the video sequences encoded by using the standard JM9.8 and the proposed approach (both under a very low target bit rate of 22 kbps) are available on the internet for comparison purposes [47].

### 4.4. Objective Quality Comparisons with State-of-the-Art Encoders on JM9.8

In this subsection, the proposed approach will be compared with the standard JM9.8 software as well as the state-of-the-art H.264/AVC encoder in [20] in terms of the actual bit rate and PSNR. The reason for running the comparisons on JM9.8 was just because the algorithm in [20] was implemented on JM9.8. In order to have a fair comparison with the approach in [20], we implemented the proposed algorithm on JM9.8 and had all the parameter settings identical to those of in [20]. The set of parameters used are listed in the previous subsection. In this experiment, three QCIF video sequences, “Foreman”, “Miss America”, and “Mother-Daughter”, as well as three CIF video sequences, “Foreman”, “Akiyo”, and “Mother-Daughter”, were used for comparisons.

The comparison results for the QCIF video sequences are shown in Table 4 and those for the CIF video sequences are shown in Table 5. Since comparison results for CIF video sequences are not reported in [20], only comparisons between the proposed approach and the standard JM9.8 are listed in Table 5. It should be noted that the actual bit rate was only an approximate value of the target bit rate in most of the video coding or transcoding systems. It was hard to match the target bit rate exactly. Therefore, direct comparisons between reported PSNRs among different approaches would have been unfair. For this, we defined in this study a performance metric called “CP index (Cost–Performance index)” as below:

(7) $$\mathrm{CP}\;\mathrm{index}=\frac{\mathrm{PSNR}\;}{\mathrm{Actual}\;\mathrm{Bit}\text{-}\mathrm{rate}}\;\left(\mathrm{in}\;\mathrm{dB}/\mathrm{kbps}\right).$$

Obviously, the Cost–Performance index provided a fair and clear comparison between different approaches, and an algorithm with a larger CP index was preferred.

As can be seen in Table 4, the proposed approach had a better CP index than that in [20] for the QCIF test video “Foreman” under a target bit rate of 19 kbps. For the second QCIF test video “Miss America”, the proposed approach exhibited the best CP index, even better than that obtained by the standard JM9.8, under the target bit rate of 68 kbps. For the third QCIF test video “Mother-Daughter”, a better CP index could be obtained by using the proposed approach than that by [20] among all three kinds of target bit rates, i.e., 11 kbps, 22 kbps, and 118 kbps. In addition, the CP index obtained by using the proposed approach was even better than that by the standard JM9.8 under the target bit rate at 118 kbps.

The comparison results for the CIF test video sequences are shown in Table 5. For comparison purposes, we list in the fifth, eighth, and eleventh columns of Table 5 the difference between the CP index obtained by using the proposed approach and that by the standard JM9.8. As we can see in Table 5, the proposed approach had a better CP index over the standard JM9.8 with the test video “Foreman” under a target bit rate of 64 kbps and the test video “Akiyo” under a target bit rate of 68 kbps.

It should be noted that the bit rate expense and the PSNR obtained in Table 4 and Table 5 were calculated with the whole image frame, but not specifically for the ROI. In certain cases, the performance (in terms of CP index) of the proposed approach was superior to that of the JM9.8 reference software and that in [20]. For those cases where the proposed approach had inferior CP index results compared to JM9.8 and [20], we noticed that the differences in the CP indices were quite trivial. This observation in conjunction with the subjective and objective comparisons in Section 4.3 indicated that the proposed approach could have limited bit resources concentrated on those ROIs and could thus enhance the visual quality of ROIs effectively, but without degrading the overall PSNR at all.

### 4.5. Comparisons with Standard JM18.4 Software

In this study, we implemented the proposed algorithm on the latest H.264/AVC reference software, JM18.4 [32]. We compared the bit rate expense, PSNR, and CP index of the proposed approach with those of JM18.4. The three QCIF video sequences “Foreman”, “Miss America”, and “Mother-Daughter” and the three CIF video sequences “Foreman”, “Akiyo”, and “Mother-Daughter” were used for this experiment. For this experiment, the parameter settings for JM18.4 are listed below.

1. RD Optimization: on
2. Entropy coding: CABAC
3. SearchRange: 16
4. NumberReferenceFrames: 2
5. NumberBFrames: 0
6. RateControlEnable: 1

The comparison results with JM18.4 are shown in Table 6 (for QCIF videos) and Table 7 (for CIF videos), respectively. For ease of comparison, we also show in the fifth, eighth, and eleventh columns of Table 6 and Table 7 the difference in the CP indices between the proposed approach and JM18.4. As can be seen in Table 6 and Table 7, the results for the CP index obtained by using the proposed approach were quite close to those obtained by using JM18.4, meaning that the proposed approach could boost the visual quality of ROIs, but without degrading the overall PSNR of the video sequence.

### 4.6. Run-Time Performance on JM9.8 and JM18.4

Finally, we compared the coding times of the proposed approach with those of standard JM reference software on JM9.8 and JM18.4 to evaluate the run-time performance of the proposed approach. The comparison results obtained by running on JM9.8 and JM18.4 are shown in Table 8 and Table 9, respectively. For comparison purposes, we also show in the sixth, ninth, and twelfth columns of Table 8 and Table 9 the percentage increase in execution time between the proposed approach and the standard JM reference software by the following equation:

(8) $$\%\;\mathrm{Increased}=\frac{{\mathrm{RunTime}}_{Proposed}-{\mathrm{RunTime}}_{JM}}{{\mathrm{RunTime}}_{JM}}.$$

For the QCIF test video sequence “Foreman” in Table 8, we can see that the coding times of the proposed approach were less than those of JM9.8 under the target bit rates of 64 kbps and 244 kbps. For most of the cases, we can see in Table 8 and Table 9 that there was only a slight increase in execution times, around 3% to 10%, for the proposed approach. Therefore, a very good trade-off between the visual quality enhancement on regions of interest and the computational complexity could be obtained with the proposed approach, which again demonstrates the superiority of the proposed perception-based dynamic QP adjustment strategy.

## 5. Limitations and Future Work

In this section, the limitations and future work of the proposed system are addressed. We begin by examining the compatibility of the proposed algorithm with CPDT transcoders, along with potential limitations and scenarios where the method may not be applicable. This is followed by a discussion of possible failure cases. Next, we explore various strategies to further reduce the computational complexity of the proposed algorithm. Finally, we outline future work, with a particular focus on incorporating SSIM and VMAF as perceptual quality metrics for more comprehensive visual quality assessment.

### 5.1. Transcoding Integration and Limitations

As stated in Section 3.4, the proposed algorithm can be integrated into the CPDT (Cascaded Pixel Domain Transcoder) framework and operates within the macroblock re-quantization stage without modifying the H.264 bitstream syntax. This allows full compatibility with existing decoders and streaming protocols. Nonetheless, some limitations are acknowledged:

A. Impact on End-to-End Latency
  The proposed perception-based QP control is implemented at the macroblock level during re-quantization. As the proposed method involves region-of-interest (ROI) analysis prior to QP reassignment, it introduces a slight additional delay in the transcoding process. This added latency, measured at approximately 3–10%, is generally acceptable for offline or buffered applications but may pose a concern for ultra-low-latency live-streaming scenarios.
B. Streaming Protocol Compatibility
  Our method operates entirely within the pixel-domain transcoding stage (CPDT), without modifying the bitstream syntax or introducing any non-standard metadata. Therefore, the output video remains fully compliant with existing H.264/AVC decoders and compatible with standard streaming protocols such as RTSP, HLS, or MPEG-DASH. That said, the additional transcoding step may require re-buffering or increased segment preparation time in certain adaptive streaming implementations.
C. Potential Limitations and Non-Applicable Scenarios
  The proposed method is most effective in scenarios where faces or motion regions are perceptually important and where some transcoding latency can be tolerated. It may not be applicable in applications that demand frame-level latency (e.g., interactive video conferencing) or in content types without clear perceptual saliency (e.g., screen-captured content, static slides, or surveillance with minimal motion). In such cases, the cost–benefit trade-off of the ROI-based re-quantization may not justify the added processing.

### 5.2. Failure Case Analysis

To further enhance the robustness and transparency of the proposed method and clarify the conditions under which it remains effective, we identify and analyze representative failure cases as follows:

A. Face Detection Failures:
  The Viola–Jones classifier may fail in detecting faces with non-frontal poses, strong lighting variations, or partial occlusions. In such cases, inaccurate ROI detection can result in inappropriate QP assignments, degrading visual quality in perceptually important regions.
B. Non-Salient Content Types:
  In scenarios such as static slides, screen recordings, or low-activity surveillance footage, the lack of perceptual saliency may cause the ROI detection mechanism to either fail to identify any meaningful regions or to assign QP levels uniformly, resulting in negligible visual improvement despite added processing.
C. False Positives from Motion Detection:
  Background motion (e.g., moving trees, passing cars) may be misclassified as perceptually important due to high motion vector magnitudes. This can lead to inefficient bit rate allocation and loss of quality in more relevant regions, such as faces.
D. Real-Time Constraints:
  Although not a failure of functionality, the method may not be suitable in real-time systems with strict latency requirements, as the added 3–10% computational cost could affect responsiveness.

These failure scenarios highlight the limitations of the proposed method. By acknowledging these scenarios, we aim to provide a realistic perspective on the method’s practical boundaries and identify areas for further refinement in future work.

### 5.3. Potential Strategies for Reducing Computational Complexity

As noted in this manuscript, the proposed method incurs a marginal runtime increase of approximately 3% to 10% due to additional operations associated with ROI classification and adaptive QP control. To address this computational overhead and enhance the system’s suitability for real-time and embedded applications, we propose several optimization strategies. These approaches are not only practically motivated but also grounded in the characteristics of the H.264/AVC encoding process and the design of our perception-based QP control mechanism. The proposed strategies are summarized below:

A. Reduced ROI Evaluation Frequency
  Instead of performing ROI analysis (including face and motion detection) on every frame, we propose a content-adaptive update strategy based on encoding parameters readily available from the H.264/AVC encoder:
  - Skip Mode Ratio: For example, if more than 80% of macroblocks in the current frame are encoded in Skip Mode (i.e., no residuals or motion compensation is needed), this suggests scene stability. In such cases, ROI analysis can be safely skipped for that frame.
  - Motion Vector Variability: We measure the frame-to-frame difference in motion vector magnitude histograms. If the histogram remains stable between two consecutive frames, this implies little change in motion saliency, and ROI re-analysis can be avoided.

The above approaches reduce per-frame processing without compromising the temporal accuracy of ROI selection and align well with the temporal redundancy principle in video encoding.

B. Simplified Motion Analysis
  To reduce the arithmetic complexity involved in computing motion vector magnitudes for macroblocks, we replace costly floating-point operations with hardware-efficient alternatives:
  - L1 Norm Approximation: Replace the Euclidean norm ($\sqrt{{MV}_{x}^{2}+{MV}_{y}^{2}}$) with the Manhattan distance ($\left|{MV}_{x}\right|+\left|{MV}_{y}\right|$), avoiding multiplication and square root operations.
  - Fixed Thresholding: Instead of calculating the average motion vector magnitude per frame, we use an empirically determined fixed threshold (e.g., 4 MV units for CIF resolution) to classify motion-intensive regions.
  - Quantized MV Binning: Motion vector magnitudes are quantized into discrete bins (e.g., 0–1, 2–3, 4–5, etc.) and mapped to pre-defined ROI weights via a lookup table. This removes the need for real-time numerical comparisons.

These techniques preserve the effectiveness of motion-based ROI detection while substantially reducing per-frame arithmetic cost and overhead.

C. Hardware-Level Optimizations
  To further improve performance on embedded platforms such as the Raspberry Pi 4 Model B, low-level optimizations can be applied:
  - NEON SIMD Instructions: Utilize ARM NEON SIMD instructions to accelerate macroblock-level pixel processing and QP assignment logic.
  - Fixed-point Arithmetic: Where applicable, convert floating-point operations (e.g., ROI weighting, QP adjustment calculations) to fixed-point approximations to take advantage of hardware-supported integer math.

These optimizations are especially effective in platforms lacking dedicated floating-point units and align with the design principles of low-power video systems.

### 5.4. Incorporating Perceptual Quality Metrics in Future Evaluations

To further enhance the comprehensiveness of performance evaluation, future work will incorporate perceptual quality metrics such as the Structural Similarity Index (SSIM) and Video Multi-Method Assessment Fusion (VMAF). These metrics provide a more human-aligned assessment of visual quality, capturing structural distortions and temporal artifacts that may not be fully reflected by traditional metrics like PSNR.

In particular, SSIM will be used to assess local luminance and contrast preservation, while VMAF, developed by Netflix, offers a machine learning-based fusion of multiple perceptual indicators. These metrics are especially valuable in low-bit-rate scenarios, where visual quality degradation is more perceptible.

The integration of SSIM and VMAF will require additional processing steps such as frame format conversion and temporal alignment. These implementations are planned as part of our future extensions and are expected to provide a more holistic view of the algorithm’s impact on perceived video quality.

## 6. Conclusions

In this paper, we proposed a perception-based visual quality enhancement algorithm for the H.264/AVC video coding system. The algorithm targets regions that are most sensitive to the human visual system, specifically areas containing human faces and regions exhibiting high motion activity. To accurately identify these regions of interest (ROIs), macroblocks containing human faces are detected using a cascade of AdaBoost classifiers, while those with significant motion are identified by comparing their motion intensity to the average motion intensity of the preceding reference frame. Once the ROIs are determined, a four-level dynamic quantization parameter (QP) adjustment strategy is applied at the macroblock level, enabling more bits to be allocated to perceptually important areas. The experimental results demonstrate that the proposed method significantly improves both subjective visual quality and objective PSNR in ROI regions, compared to the standard JM reference software and other state-of-the-art approaches, under the same target bit rate constraint. It is worth noting that the proposed method achieves this enhancement with only a slight increase in computational complexity, making it highly suitable for real-time video applications in resource-constrained environments.

## Figures and Tables

**Table 2:** The test sequences corresponding to each bitrate setting.

Image Resolution Target Bitrates	QCIF Test Sequences	CIF Test Sequences
244 kbps	Foreman (15 fps)	Foreman (15 fps)
118 kbps	Mother-Daughter (30 fps)	Mother-Daughter (30 fps)
68 kbps	MissAmerica (30 fps)	Akiyo (30 fps)
64 kbps	Foreman (15 fps)	Foreman (15 fps)
22 kbps	Mother-Daughter (30 fps)	Mother-Daughter (30 fps)
19 kbps	Foreman (15 fps)	Foreman (15 fps)
11 kbps	MissAmerica (30 fps) Mother-Daughter (30 fps)	Akiyo (30 fps) Mother-Daughter (30 fps)
8 kbps	MissAmerica (30 fps)	Akiyo (30 fps)

**Table 3:** Comparison of Viola-Jones (Haar Cascade Classifier) and MobileNetV1-SSD (INT8) for Face Detection on Edge Devices.

Metric	Viola-Jones (Haar)	MobileNetV1-SSD (INT8)	Remarks
Input Resolution	CIF (352 × 288)	300 × 300	
FLOPs	2 M	570 M	Inference per image
Model Size	<1 MB	6.8 MB	
RAM Usage	20 MB	30 MB	Includes model, input, buffer memory
FPS	Approx. 15	Approx. 8	Estimated on Raspberry Pi 4
Detection Rate	70%	88%	
Precision	0.85	0.87	With IoU = 0.5
Recall	0.68	0.85	With IoU = 0.5
Applicable Scenarios	Frontal face detection in real-time low-power systems	Robust detection across multiple views, occlusion and lighting conditions	
Platform Compatibility	High	High	

**Table 4:** Bitrate and PSNR performance on JM9.8 with QCIF test video sequence.

**QCIF Video**	**Metric**	**Target Bitrate 19 kb/s**			**Target Bitrate 64 kb/s**			**Target Bitrate 244 kb/s**		
	**Scheme**	**JM9.8**	**Proposed**	**Ref. [20]**	**JM9.8**	**Proposed**	**Ref. [20]**	**JM9.8**	**Proposed**	**Ref. [20]**
Foreman 15 fps	Actual Bitrates (Kbps)	19.03	19.20	19.04	64.02	64.30	64.03	244.00	244.46	244.02
	PSNR (dB)	29.58	27.84	27.10	36.11	33.13	33.86	43.25	38.72	41.07
	PSNR/Bitrate	1.554	1.450	1.423	0.564	0.515	0.529	0.177	0.158	0.168
**QCIF Video**	**Metric**	**Target Bitrate 8 kb/s**			**Target Bitrate 11 kb/s**			**Target Bitrate 68 kb/s**		
	**Scheme**	**JM9.8**	**Proposed**	**Ref. [20]**	**JM9.8**	**Proposed**	**Ref. [20]**	**JM9.8**	**Proposed**	**Ref. [20]**
Miss America 30 fps	Actual Bitrates (Kbps)	8.06	8.59	8.04	11.07	11.16	11.04	68.16	68.37	68.03
	PSNR (dB)	32.98	32.95	32.82	35.15	34.53	34.61	42.40	42.74	42.23
	PSNR/Bitrate	4.091	3.836	4.082	3.175	3.094	3.135	0.622	0.625	0.621
**QCIF Video**	**Metric**	**Target Bitrate 11 kb/s**			**Target Bitrate 22 kb/s**			**Target Bitrate 118 kb/s**		
	**Scheme**	**JM9.8**	**Proposed**	**Ref. [20]**	**JM9.8**	**Proposed**	**Ref. [20]**	**JM9.8**	**Proposed**	**Ref. [20]**
Mother-Daughter 30 fps	Actual Bitrates (Kbps)	11.04	11.25	11.05	22.05	22.15	22.05	118.11	118.40	118.07
	PSNR (dB)	31.62	31.54	28.74	34.31	34.03	30.85	42.09	42.30	37.98
	PSNR/Bitrate	2.864	2.804	2.600	1.556	1.536	1.399	0.356	0.357	0.322

**Table 5:** Bitrate and PSNR performance on JM9.8 with CIF test video sequence.

**CIF Video**	**Metric**	**Target Bitrate 19 kb/s**			**Target Bitrates 64 kb/s**			**Target Bitrates 244 kb/s**		
	**Scheme**	**JM9.8**	**Proposed**	**Difference** **JM-Proposed**	**JM9.8**	**Proposed**	**Difference** **JM-Proposed**	**JM9.8**	**Proposed**	**Difference** **JM-Proposed**
Foreman 15 fps	Actual Bitrates (Kbps)	19.02	19.29	−0.27	64.05	64.06	−0.01	244.06	244.11	−0.05
	PSNR (dB)	24.66	24.68	−0.02	31.29	31.69	−0.40	37.00	37.11	−0.11
	PSNR/Bitrate	1.297	1.279	0.018	0.489	0.495	−0.006	0.152	0.152	0.000
**CIF Video**	**Metric**	**Target Bitrate 8 kb/s**			**Target Bitrates 11 kb/s**			**Target Bitrates 68 kb/s**		
	**Scheme**	**JM9.8**	**Proposed**	**Difference** **JM-Proposed**	**JM9.8**	**Proposed**	**Difference** **JM-Proposed**	**JM9.8**	**Proposed**	**Difference** **JM-Proposed**
Akiyo 30 fps	Actual Bitrates (Kbps)	8.22	8.66	−0.44	11.04	11.62	−0.58	68.12	68.28	−0.16
	PSNR (dB)	29.57	29.85	−0.28	31.74	32.11	−0.37	38.59	39.39	−0.80
	PSNR/Bitrate	3.597	3.447	0.150	2.875	2.763	0.112	0.567	0.577	−0.010
**CIF Video**	**Metric**	**Target Bitrate 11 kb/s**			**Target Bitrates 22 kb/s**			**Target Bitrates 118 kb/s**		
	**Scheme**	**JM9.8**	**Proposed**	**Difference** **JM-Proposed**	**JM9.8**	**Proposed**	**Difference** **JM-Proposed**	**JM9.8**	**Proposed**	**Difference** **JM-Proposed**
Mother-Daughter 30 fps	Actual Bitrates (Kbps)	11.05	11.37	−0.32	22.06	22.38	−0.32	118.18	118.19	−0.01
	PSNR (dB)	29.17	29.76	−0.59	31.92	31.96	−0.04	38.51	38.21	0.30
	PSNR/Bitrate	2.640	2.617	0.0023	1.447	1.428	0.019	0.326	0.323	0.003

**Table 6:** Bitrate and PSNR performance on JM18.4 with QCIF test video sequences.

**QCIF Video**	**Metric**	**Target Bitrate 19 kb/s**			**Target Bitrate 64 kb/s**			**Target Bitrate 244 kb/s**		
	**Scheme**	**JM18.4**	**Proposed**	**Difference** **JM-Proposed**	**JM18.4**	**Proposed**	**Difference** **JM-Proposed**	**JM18.4**	**Proposed**	**Difference** **JM-Proposed**
Foreman 15 fps	Actual Bitrates (Kbps)	19.02	18.97	0.05	64.00	64.05	−0.05	243.91	243.85	0.06
	PSNR (dB)	29.98	29.89	0.09	36.63	36.56	0.07	43.82	43.75	0.07
	PSNR/Bitrate	1.576	1.576	0.000	0.572	0.571	0.001	0.180	0.179	0.001
**QCIF Video**	**Metric**	**Target Bitrate 8 kb/s**			**Target Bitrate 11 kb/s**			**Target Bitrate 68 kb/s**		
	**Scheme**	**JM18.4**	**Proposed**	**Difference** **JM-Proposed**	**JM18.4**	**Proposed**	**Difference** **JM-Proposed**	**JM18.4**	**Proposed**	**Difference** **JM-Proposed**
Miss America 30 fps	Actual Bitrates (Kbps)	10.78	11.45	−0.67	13.18	14.18	−1.00	67.98	67.93	0.05
	PSNR (dB)	35.03	35.36	−0.33	36.18	36.28	−0.10	42.96	42.82	0.14
	PSNR/Bitrate	3.250	3.088	0.162	2.745	2.559	0.186	0.632	0.630	0.002
**QCIF Video**	**Metric**	**Target Bitrate 11 kb/s**			**Target Bitrate 22 kb/s**			**Target Bitrate 118 kb/s**		
	**Scheme**	**JM18.4**	**Proposed**	**Difference** **JM-Proposed**	**JM18.4**	**Proposed**	**Difference** **JM-Proposed**	**JM18.4**	**Proposed**	**Difference** **JM-Proposed**
Mother-Daughter 30 fps	Actual Bitrates (Kbps)	12.06	12.16	−0.10	22.12	22.65	−0.53	118.36	118.55	−0.19
	PSNR (dB)	32.55	32.57	−0.02	35.06	34.99	0.07	42.58	42.40	0.18
	PSNR/Bitrate	2.699	2.678	0.021	1.585	1.545	0.040	0.360	0.358	0.002

**Table 7:** Bitrate and PSNR performance on JM18.4 with CIF test video sequences.

**CIF Video**	**Metric**	**Target Bitrate 19 kb/s**			**Target Bitrate 64 kb/s**			**Target Bitrate 244 kb/s**		
	**Scheme**	**JM18.4**	**Proposed**	**Difference** **JM-Proposed**	**JM18.4**	**Proposed**	**Difference** **JM-Proposed**	**JM18.4**	**Proposed**	**Difference** **JM-Proposed**
Foreman 15 fps	Actual Bitrates (Kbps)	34.73	39.31	−4.58	64.12	64.08	0.04	243.65	243.82	−0.17
	PSNR (dB)	28.84	29.30	−0.46	31.87	31.75	0.12	37.42	37.36	0.06
	PSNR/Bitrate	0.830	0.745	0.085	0.497	0.495	0.032	0.154	0.153	0.001
**CIF Video**	**Metric**	**Target Bitrate 8 kb/s**			**Target Bitrate 11 kb/s**			**Target Bitrate 68 kb/s**		
	**Scheme**	**JM18.4**	**Proposed**	**Difference** **JM-Proposed**	**JM18.4**	**Proposed**	**Difference** **JM-Proposed**	**JM18.4**	**Proposed**	**Difference** **JM-Proposed**
Akiyo 30 fps	Actual Bitrates (Kbps)	14.51	19.30	−4.79	14.51	19.30	−4.79	67.98	67.96	0.02
	PSNR (dB)	33.52	34.75	−1.23	33.52	34.75	−1.23	39.73	39.20	0.53
	PSNR/Bitrate	2.310	1.800	0.510	2.310	1.800	0.510	0.584	0.577	0.007
**CIF Video**	**Metric**	**Target Bitrate 11 kb/s**			**Target Bitrate 22 kb/s**			**Target Bitrate 118 kb/s**		
	**Scheme**	**JM18.4**	**Proposed**	**Difference** **JM-Proposed**	**JM18.4**	**Proposed**	**Difference** **JM-Proposed**	**JM18.4**	**Proposed**	**Difference** **JM-Proposed**
Mother-Daughter 30 fps	Actual Bitrates (Kbps)	18.84	22.48	−3.64	24.01	24.92	−0.91	118.17	118.21	−0.04
	PSNR (dB)	32.02	32.62	−0.60	32.84	32.81	0.03	39.40	39.06	0.34
	PSNR/Bitrate	1.700	1.451	0.249	1.368	1.317	0.051	0.333	0.330	0.003

**Table 8:** Run-time performance evaluation with QCIF and CIF test video sequences on JM9.8.

QCIF Video	Foreman 15 fps	**Target Bitrate**	**Target Bitrate 19 kb/s**			**Target Bitrate 64 kb/s**			**Target Bitrate 244 kb/s**		
		**Scheme**	**JM9.8**	**Proposed**	**% Increased**	**JM9.8**	**Proposed**	**% Increased**	**JM9.8**	**Proposed**	**% Increased**
		**RunTime (in Second)**	376.73	378.24	0.40%	430.48	417.39	−3%	534.08	477.06	−10.70%
	Miss America 30 fps	**Target Bitrate**	**Target Bitrate 8 kb/s**			**Target Bitrate 11 kb/s**			**Target Bitrate 68 kb/s**		
		**Scheme**	**JM9.8**	**Proposed**	**% Increased**	**JM9.8**	**Proposed**	**% Increased**	**JM9.8**	**Proposed**	**% Increased**
		**RunTime (in Second)**	169.54	178.70	5.40%	173.86	180.79	4%	195.05	206.01	5.60%
	Mother-Daughter 30 fps	**Target Bitrate**	**Target Bitrate 11 kb/s**			**Target Bitrate 22 kb/s**			**Target Bitrate 118 kb/s**		
		**Scheme**	**JM9.8**	**Proposed**	**% Increased**	**JM9.8**	**Proposed**	**% Increased**	**JM9.8**	**Proposed**	**% Increased**
		**RunTime (in Second)**	352.76	362.75	2.80%	371.49	381.64	2.70%	445.60	465.91	4.60%
CIF Video	Foreman 15 fps	**Target Bitrate**	**Target Bitrate 19 kb/s**			**Target Bitrate 64 kb/s**			**Target Bitrate 244 kb/s**		
		**Scheme**	**JM9.8**	**Proposed**	**% Increased**	**JM9.8**	**Proposed**	**% Increased**	**JM9.8**	**Proposed**	**% Increased**
		**RunTime (in Second)**	1400.90	1482.68	5.80%	1509.80	1581.95	4.80%	1696.96	1760.39	3.75%
	Akiyo 30 fps	**Target Bitrate**	**Target Bitrate 8 kb/s**			**Target Bitrate 11 kb/s**			**Target Bitrate 68 kb/s**		
		**Scheme**	**JM9.8**	**Proposed**	**% Increased**	**JM9.8**	**Proposed**	**% Increased**	**JM9.8**	**Proposed**	**% Increased**
		**RunTime (in Second)**	1290.65	1401.30	8.60%	1322.32	1412.32	6.80%	1503.19	1588.81	5.70%
	Mother-Daughter 30 fps	**Target Bitrate**	**Target Bitrate 11 kb/s**			**Target Bitrate 22 kb/s**			**Target Bitrate 118 kb/s**		
		**Scheme**	**JM9.8**	**Proposed**	**% Increased**	**JM9.8**	**Proposed**	**% Increased**	**JM9.8**	**Proposed**	**% Increased**
		**RunTime (in Second)**	1340.95	1424.93	6.30%	1388.86	1459.98	5.10%	1526.84	1580.95	3.50%

**Table 9:** Run-time performance evaluation with QCIF and CIF test video sequences on JM18.4.

QCIF Video	Foreman 15 fps	**Target Bitrate**	**Target Bitrate 19 kb/s**			**Target Bitrate 64 kb/s**			**Target Bitrate 244 kb/s**		
		**Scheme**	**JM18.4**	**Proposed**	**% Increased**	**JM18.4**	**Proposed**	**% Increased**	**JM18.4**	**Proposed**	**% Increased**
		**RunTime (in Second)**	262.16	274.36	4.70%	287.67	299.65	4.20%	329.02	342.23	4.00%
	Miss America 30 fps	**Target Bitrate**	**Target Bitrate 8 kb/s**			**Target Bitrate 11 kb/s**			**Target Bitrate 68 kb/s**		
		**Scheme**	**JM18.4**	**Proposed**	**% Increased**	**JM18.4**	**Proposed**	**% Increased**	**JM18.4**	**Proposed**	**% Increased**
		**RunTime (in Second)**	105.53	114.74	8.70%	107.25	116.14	8.30%	115.99	124.99	7.80%
	Mother-Daughter 30 fps	**Target Bitrate**	**Target Bitrate 11 kb/s**			**Target Bitrate 22 kb/s**			**Target Bitrate 118 kb/s**		
		**Scheme**	**JM18.4**	**Proposed**	**% Increased**	**JM18.4**	**Proposed**	**% Increased**	**JM18.4**	**Proposed**	**% Increased**
		**RunTime (in Second)**	221.68	234.70	5.90%	234.11	246.03	5.10%	260.80	274.65	5.30%
CIF Video	Foreman 15 fps	**Target Bitrate**	**Target Bitrate 19 kb/s**			**Target Bitrate 64 kb/s**			**Target Bitrate 244 kb/s**		
		**Scheme**	**JM18.4**	**Proposed**	**% Increased**	**JM18.4**	**Proposed**	**% Increased**	**JM18.4**	**Proposed**	**% Increased**
		**RunTime (in Second)**	952.50	1025.92	7.70%	982.54	1050.12	6.90%	1085.37	1157.42	6.60%
	Akiyo 30 fps	**Target Bitrate**	**Target Bitrate 8 kb/s**			**Target Bitrate 11 kb/s**			**Target Bitrate 68 kb/s**		
		**Scheme**	**JM18.4**	**Proposed**	**% Increased**	**JM18.4**	**Proposed**	**% Increased**	**JM18.4**	**Proposed**	**% Increased**
		**RunTime (in Second)**	795.62	877.65	10.30%	795.60	876.63	10.20%	850.86	918.82	8.00%
	Mother-Daughter 30 fps	**Target Bitrate**	**Target Bitrate 11 kb/s**			**Target Bitrate 22 kb/s**			**Target Bitrate 118 kb/s**		
		**Scheme**	**JM18.4**	**Proposed**	**% Increased**	**JM18.4**	**Proposed**	**% Increased**	**JM18.4**	**Proposed**	**% Increased**
		**RunTime (in Second)**	802.54	881.92	9.90%	808.80	886.72	9.60%	893.76	967.14	8.20%

## Data Availability

The test video sequences used in this study are available from the following public website: https://media.xiph.org/video/derf (accessed on 17 April 2025).

## Abbreviations

The following abbreviations are used in this manuscript:

AdaBoost	Adaptive Boosting
AVC	Advanced Video Coding
CCTV	Closed-Circuit Television
CDDT	Cascade DCT Domain Transcoder
CIF	Common Intermediate Format
CPDT	Cascade Pixel Domain Transcoder
HD	High Definition
HEVC	High-Efficiency Video Coding
IoT	Internet of Things
JM	Joint Model
JND	Just Noticeable Distortion
MB	Macroblock
NZTC	Non-zero Transform Coefficients
PSNR	Peak Signal-to-Noise Ratio
QCIF	Quarter Common Intermediate Format
QP	Quantization Parameter
RDO	Rate Distortion Optimization
ROI	Region of Interest
VVC	Versatile Video Coding
